# Surface characterization and corrosion resistance properties with nitriding time of carbon steel treated in V_2_O_5_-added salt bath

**DOI:** 10.1039/d6ra03818b

**Published:** 2026-08-27

**Authors:** Song-Chun Ri, Kyong-Sik Ju

**Affiliations:** a High-Tech Research and Development Center, Kim Il Sung University Pyongyang Democratic People's Republic of Korea ks.ju1025@ryongnamsan.edu.kp

## Abstract

In this study, a nitriding salt bath containing 1 wt% V_2_O_5_ was employed for treatment of carbon steel samples for several hours (20, 40, 60, 80, 100, 120 min) at a temperature of 500 ± 10 °C and the possibility for accelerating the nitriding process was examined. The microstructure, phase composition, micro-hardness and wear resistance for the non-nitrided and nitrided samples were evaluated through general characterization. The surface corrosion properties of nitrided samples were evaluated through neutral salt water spray test, electrochemical polarization test, and electrochemical impedance spectroscopy. Fe_3_C was not observed in the nitride layers composed of Fe_3_N and Fe_4_N in the range of 10–25 µm. The values of micro-hardness for the nitride layers were not linearly related to nitriding time, reaching a maximum of 627 HV_0.1_. The corrosion resistance of the nitrided samples with V_2_O_5_ was improved significantly compared to the untreated sample.

## Abstract 2

A nitriding salt bath containing 1 wt% V_2_O_5_ was employed for treatment of carbon steel samples for several hours at a temperature of 500 ± 10 °C and the possibility for accelerating the nitriding process was examined.

## Graphical abstract

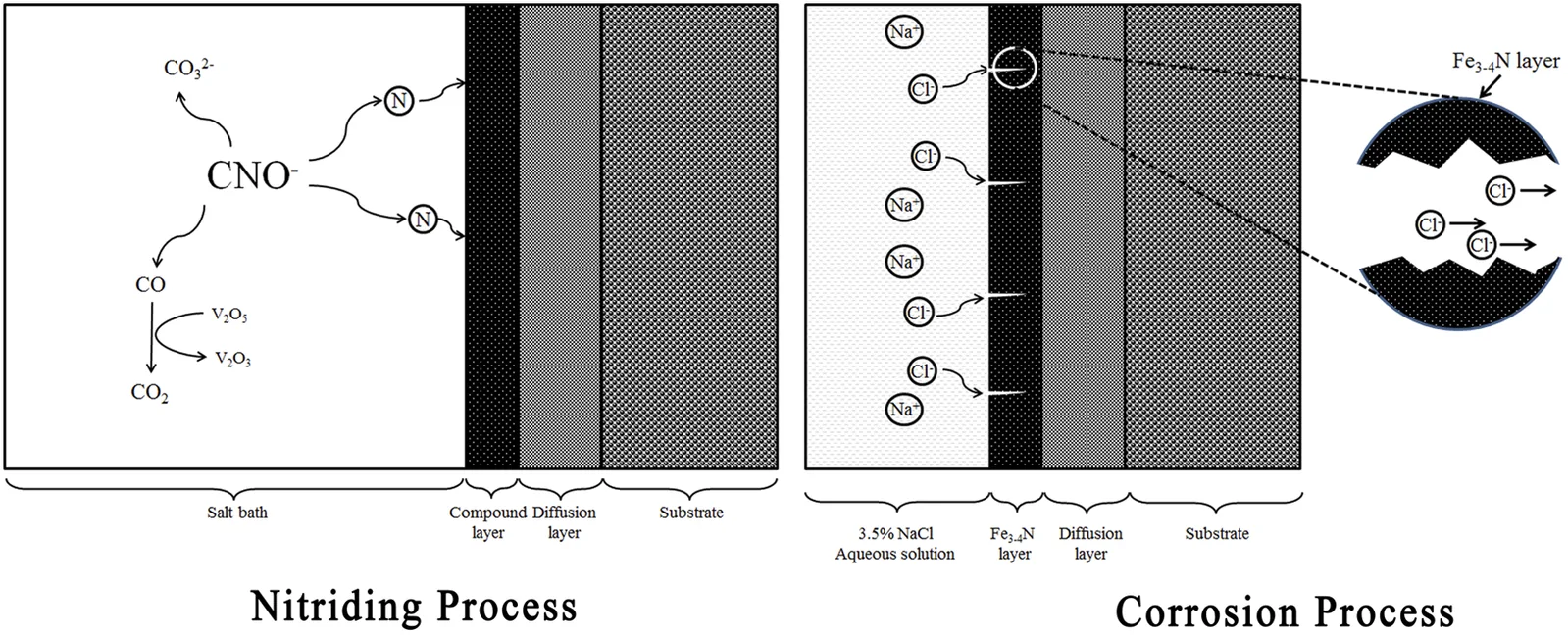

## 1. Introduction

Nitriding techniques, which are surface treatment techniques for various steel and stainless steel materials, have been widely used to improve the hardness, fatigue strength, wear resistance and corrosion resistance of materials. The salt bath nitriding process has many advantages over gas nitriding and plasma nitriding, such as low temperature, short processing time, high degree of dimensional stability, and reproducibility.^1–8,25,28,29^ Furthermore, it is suggested that the surface corrosion resistance provided by the QPQ technique, which combines salt bath nitriding and salt bath oxidation processes, is better than that of hard chromium or nickel plating.^9–14^ Using the QPQ process, the enhancement of fatigue resistance, wear resistance and corrosion resistance for various grades of metallic materials can be achieved.^15–22,31,32,35^ The QPQ process mainly consists of six steps: (1) degreasing (2) preheating (3) nitriding (4) oxidizing (5) washing and (6) oil immersion. Nitriding and oxidizing are prone to producing nitride and oxide layers on the material surface, and are the key steps of the whole process.^26^ The outermost layer of the material after treatment consisted of Fe_3_O_4_ with good corrosion resistance, indicating that the Fe_3_O_4_ film deposited on the nitride layer is significantly affected by the nitriding conditions.^28,34^ Therefore, it is shown that the phase composition and microstructure of the nitride layer have a significant influence on fatigue resistance, wear resistance and corrosion resistance of materials after QPQ treatment.

Low carbon steel, which is cheap and has good mechanical properties, is widely used as structural steel in industry, but its application is limited by its low corrosion and wear resistance. It can be seen that if its wear and corrosion resistance are improved through the complex salt bath treatment of low carbon steel, the application area can be greatly increased. For example, if low carbon steel materials with improved corrosion resistance are used for heat exchangers operating in seawater, considerable economic benefits can be obtained.

Recently, low-temperature nitriding processes that reduce the energy consumption of nitriding processes and ensure wear resistance without degradation of hardness have been attracting interest.^18,20,23^ Furthermore, low temperature nitriding can reduce the distortion of precision parts. Unfortunately, low-temperature liquid nitriding is hindered by the high natural melting point of the most composition of liquid nitriding.^27^ Also, the lower the nitriding temperature, the longer the nitriding time can be. Therefore, it is of interesting and practical importance to develop a method to ensure the integrity of the nitride layer and shorten the nitriding time at low temperature. It also provides the potential to reduce the internal tissue changes of steel and may contribute to the improvement of the treatment efficiency in the salt bath.

Accelerating nitriding is an interesting topic in chemical heat treatment of ferrous parts. The thermochemical treatment with rare earths has been investigated for the past several decades and the catalytic and micro-alloying effects of rare earth in gas nitriding, plasma nitriding and ion nitriding have been investigated.^40–43^ The involvement of rare earth elements can help the interstitial carbon and nitrogen diffuse into the work-piece deeper, and at the same time improve the microstructure and the properties of the modified layer.^38^ In addition, the study of QPQ rare earth nitriding using yttrium was also carried out.^39^ However, the use of rare earths increases the processing cost and limits their application, so it is necessary to explore compounds that can replace rare earths. Few studies have been carried out to accelerate nitriding using V_2_O_5_, which is widely used as a catalyst in industry.

Thus, the aim of this study is to investigate the effect of nitriding time on the properties of low carbon steel samples treated in V_2_O_5_-added salt bath. Through investigation, the formation and development of nitride layer in V_2_O_5_-added salt bath was examined. Nitriding was carried out for several hours (20, 40, 60, 80, 100, 120 min) at 500 ± 10 °C using a salt bath containing about 1 wt% V_2_O_5_. General characterizations were carried out by comparing the chemical composition, microstructure, hardness and wear resistance of the untreated and treated surfaces. The surface corrosion characterizations after the salt bath nitriding were examined through neutral salt spray test, electrochemical polarization test, and electrochemical impedance spectroscopy (EIS).

## 2. Experiment

### 2.1. Materials and treatments

The material used in this study was a carbon steel with chemical composition (wt%) of 0.09–0.15% C, 0.12–0.3% Si, 0.25–0.5% Mn, <0.045% P, <0.05% S, and balance Fe. The carbon steel samples had a form (10 mm × 10 mm × 2 mm) to be used to estimate morphology, micro-hardness and corrosion resistance. For all the experiments, the carbon steel samples were polished with SiC paper (240, 600, 1000) to produce a fine surface finishing, and ultrasonically cleaned with deionized water and ethanol for 5 min before use.

Similar to the salt bath nitriding,^23,27^ the salt medium for nitriding was mainly composed of NaCNO, KCNO, Na_2_CO_3_, K_2_CO_3_ and about 1 wt% of V_2_O_5_. CNO^−^ concentration in the salt was 32–35% and the ratio of K and Na in the salt bath was kept in 1 : 1. The atomic nitrogen utilized for nitriding reaction comes from the dissociation of NaCNO and KCNO. The carbon steel samples were immersed in a salt bath for several hours (20, 40, 60, 80, 100, 120 min) and then quenched in water at room temperature. The samples were carefully washed with double distilled water. All of the salt bath nitriding was carried out at 500 ± 10 °C.

### 2.2. Characterization

For the used samples, one edge of the samples was cut to 2 mm in order to obtain the cross-sections. Then, the side edge surface of the samples was polished with fine abrasive paper and was etched by using an ethanol–nitric acid (5%) solution for SEM analysis. The morphological analysis in the modified layers was performed using by Scanning Electron Microscopy (SEM, JSM-6610A). Secondary electron images were obtained at 20 kV, 1.0 nA. Elemental distribution profiles of the cross-section of the nitride layers were evaluated by energy dispersive spectrometry (EDS). The phase analysis on the modified layers was carried out using by X-ray diffraction (XRD, Rigaku Smartlab). XRD patterns were obtained in the range of 2*θ* from 10° to 70° using Cu Kα radiation (*λ* = 1.54 Å). Micro-hardness tests on cross-section of samples were performed by employing loads of 100 g and dwell times of 12 s for all the experiments. The indentations on the samples were used to prepare the Micro-hardness plots on Micro-hardness Tester (ΠMP-3). The wear tests were carried out on a MMW-1A Wear Test Machine with GCr15 steel ball (6 mm in diameter) rotated at a speed of 200 rpm and a load of 50 N against the surface of the samples for the testing time of 60 min. Before tests, both the non-nitrided and nitrided samples were cleaned with alcohol. They were cleaned and dried and were then measured using an electron analytical balance (AEG-120) with an accuracy of 0.1 mg. The average mass loss was calculated by three parallel experiments carried out under the same conditions.

Neutral brine spray tests were carried out by spraying neutral brine solution (3.5%) at 25 °C on untreated and treated samples based on ISO 9227-2006 and the initial rust-producing times of the samples was recorded. Three batches of tests were performed, and the data reported in this paper represents the average value of the three tests. Further corrosion tests were performed electrochemically at room temperature (25 °C) in a polymethyl methacrylate cell for polarization curves and AC impedance measurements. A saturated calomel electrode (SCE) was used as the reference; Pt electrode was used as the counter; specimens were used as the working electrodes. The working electrodes were prepared from the samples insulated with epoxy resin such that the area exposed to solution was 1 cm^2^. To minimize ohmic resistance, a fine Luggin capillary was placed close to the working electrode. To consider the real environment of sea water, all test solutions were not de-aerated in the cell by using an inert gas. After immersion of 30 min, the polarization curves for untreated and treated samples were conducted in 3.5% NaCl solution at a scan rate of 0.01 V s^−1^ in the range of −1.0 to −0.5 V using CHI 604E electrochemical workstation controlled by PC. The polarization curves were analyzed with CHI Software. AC impedance measurements were performed in a frequency range from 10^5^ Hz to 1 Hz after 2 h of immersion in the testing solution. The impedance data were analyzed and fitted with the simulation equivalent circuit from ZsimpWin Software.

## 3. Results and discussion

### 3.1. Morphology and elemental concentration profile

**Fig. 1:**
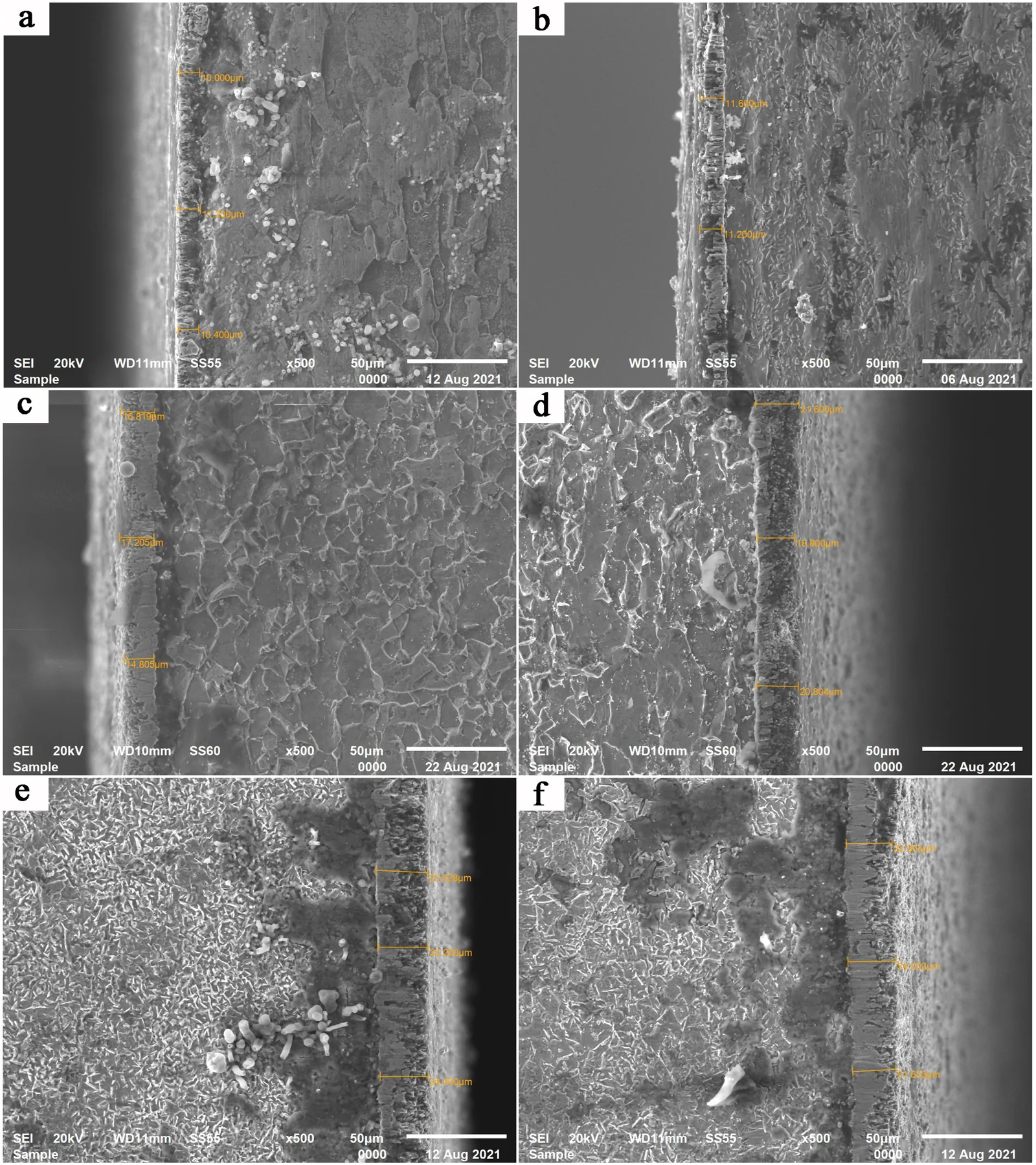
Cross-sectional secondary electron images of the samples nitrided at 500 °C for different times (a) 20 min (b) 40 min (c) 60 min (d) 80 min (e) 100 min (f) 120 min.

Fig. 1 shows the microstructures of cross-sections of the steel samples treated at 500 ± 10 °C for several hours (20, 40, 60, 80, 100 and 120 min). As can be seen from the secondary electron images shown in Fig. 1, the depth of the nitride layer is observed to be approximately 10–25 µm, reaching an average of 20 µm for a relatively short time.^23,27,30,33^ The longer the nitriding time is, the thicker the nitride layer depth is. The infiltration layers formed at the outermost of the nitrided samples were slightly etched and consisted of Fe_3_N and Fe_4_N. This indicates that the subsurface zones containing mainly α-Fe and Fe_4_N are susceptible to etching in ethanol-nitric acid (5%) solution, in contrast to the compound regions Fe_3_N produced on the surface.

**Fig. 2:**
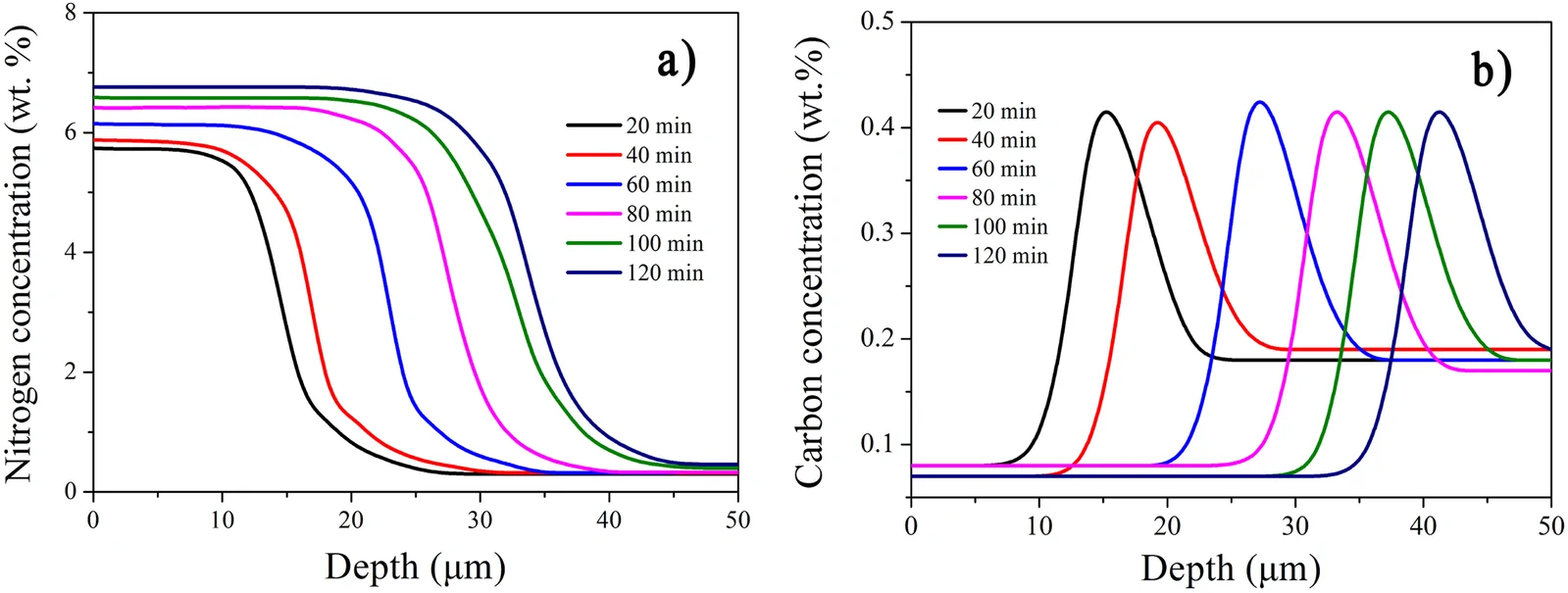
Concentration profiles of (a) nitrogen and (b) carbon as a function of depth.

Fig. 2 shows the elemental concentration profiles of nitride layers as a function of depth from the surface of samples. As shown in Fig. 2a, it can be observed the maximum of nitrogen concentration on the surface of the nitrided samples. On the other hand, it can be observed that a distribution curve of nitrogen close to the exponential decay in the range 10–25 µm, indicating the end of the compound layer. This is probably due to the formation of nitrides on the surface and the formation of Fe_3_N and Fe_4_N in the substrate. In addition, the concentration of nitrogen over the 10–25 µm range gradually decreases from the surface to the substrate, but not to the core level, meaning that nitrogen gradually diffuses from the surface to the core during the treatment.

At the beginning of nitriding, nitrogen atoms can penetrate preferentially along the grain boundaries between iron carbide and iron. The nitride layers are formed by the expansion of the nitride domains, and the concentration of carbon atoms is lower than that of the substrate layer due to the low solubility of carbon atoms in the nitride layer.^25^ As can be seen in Fig. 2b, it can be confirmed that the carbon concentration near the surface of the nitrided samples is lower than that of the substrate layer.

However, in Fig. 2b, carbon peaks with a magnitude of about 0.4 wt% can be observed in the regions where the nitrogen concentration decreases rapidly. As nitriding time increases, the position of the carbon peaks shows a tendency to shift gradually towards the substrate. This may be due to the push-effect by nitrogen.^36^ Since the solubility of carbon in the nitride region is smaller than that of the substrate, carbon atoms are pushed away from the surface by the incoming nitrogen and moved towards the substrate. Finally, carbon atoms are gradually concentrated and accumulated between the compound layer and the diffusion layer, and a carbon peak of about 0.4 wt% is recorded. Some studies have explained this phenomenon by a trapping model based on the fact that the trap binding energy of carbon is less than that of nitrogen.^37^

### 3.2. Phase composition

**Fig. 3:**
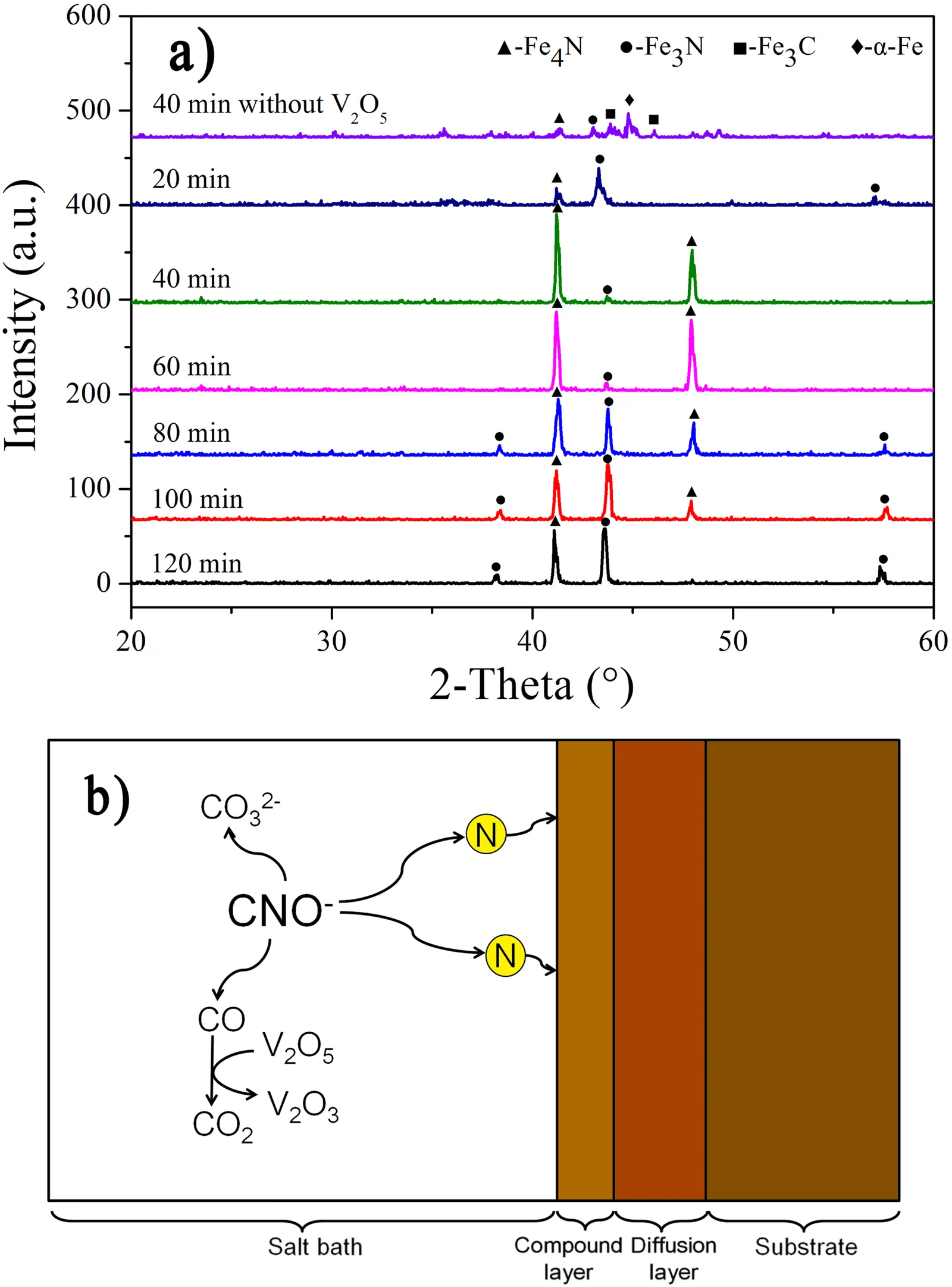
(a) XRD patterns of samples nitrided at 500 °C for different time with and without V_2_O_5_ and (b) schematic diagram of nitriding system.

The XRD patterns of the treated samples with and without V_2_O_5_ at 500 °C are shown in Fig. 3. For the treated samples with V_2_O_5_, XRD patterns with diffraction peaks corresponding to Fe_4_N and Fe_3_N phases can be observed, while no peaks corresponding to Fe_3_C or Fe are observed in the patterns. From the position and height of the peaks in these patterns, it is obvious that the phase composition of the nitride layer depends on nitriding time. The Fe_4_N and Fe_3_N peaks can be observed in all the patterns of the treated samples with V_2_O_5_. Peaks corresponding to the Fe_4_N phase for the samples nitrided for 40 and 60 min, and peaks corresponding to the Fe_4_N and Fe_3_N phases for the samples nitrided for 20, 80, 100, and 120 min can be observed. With increasing nitriding time, the intensities of the Fe_4_N and Fe_3_N peaks are both strong, however, the peak height of Fe_3_N phases becomes higher when the treating time exceeds 100 min. This is likely due to the development of the structure of Fe_3_N because of the gradual increase in nitrogen concentration in the nitride layers due to the penetration and diffusion of nitrogen atoms. This agrees well with the results obtained from the nitrogen concentration profiles (see Fig. 2a). Atomic nitrogen is generated from the decomposition of CNO^−^ ions according to the following reaction, and atomic nitrogen is adsorbed on steel samples.^26^

1 4CNO^−^ → CO_3_^2−^ + 2CN^−^ + CO + 2[N]

During the diffusion and reaction of atomic nitrogen into the steel, the layers of Fe–N solid solutions, called compound layer and diffusion layer, are produced on the surface of the samples. Compared with the steel, Fe–N layer has very high micro-hardness and excellent wear resistance.^27,34^

The peaks corresponding to Fe, Fe_3_C, Fe_4_N and Fe_3_N are included in the XRD patterns of the treated samples for 40 min without V_2_O_5_. From the peaks for Fe_3_C, it can be seen that the carbonization process proceeds simultaneously during nitriding without V_2_O_5_. According to a literature,^23^ the atomic carbons are generated from CO molecules by the decomposition of CNO^−^ and penetrated into the sample surface to form a Fe_3_C solid solution. The reaction equation is as following.

2 2CO → CO_2_ + [C]

3 Fe + 3[C] → Fe_3_C

However, peaks for iron carbide are not observed in the patterns for the treated samples with V_2_O_5_. This suggests that the generation of atomic carbons in V_2_O_5_-added salt bath is inhibited. Therefore, the decomposition reaction of CO in the reaction system containing V_2_O_5_ may be assumed as follows:

4 V_2_O_5_ + 2CO → V_2_O_3_ + 2CO_2_

By performing the reaction thermodynamic calculations at 500 °C using the HSC Chemistry 6.0 program, the Gibbs energy change (Δ*G*) is −35.293 kJ mol^−1^ for reaction (2) and −232.717 kJ mol^−1^ for reaction (4).

In addition, the comparison of the two specimens treated for the same time with and without V_2_O_5_ showed significant differences. For the treated samples without V_2_O_5_, the intensity of the peak associated with Fe–N is weak and the peak with Fe is also observed, indicating that the nitride layer is not formed sufficiently during the nitriding time of 40 min. Thus, it can be inferred that the nitride layer formed in a short time by promoting nitriding process in the V_2_O_5_-added salt bath.

### 3.3. Micro-hardness profile

**Fig. 4:**
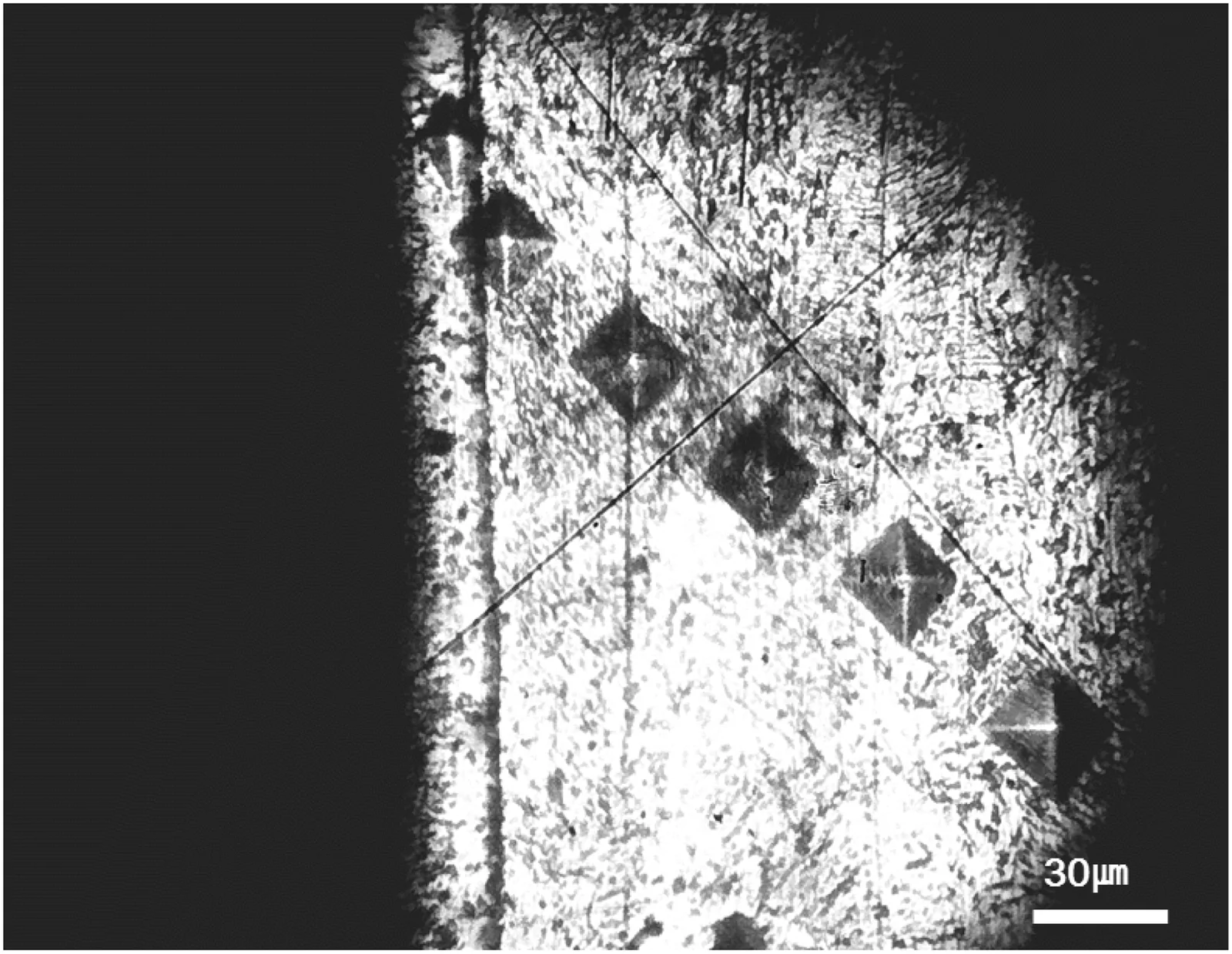
Metallograph of the indented specimen treated at 500 °C for 80 min.

The micrograph of the indentations pressed by the diamond cone in the cross section of the sample treated at 500 °C for 80 min is shown in Fig. 4. The maximum micro-hardness of the cross-section of the sample treated for 80 min is more than 612 HV _0.1_ and nearly three times that of the core 223 HV_0.1_.^26^

**Fig. 5:**
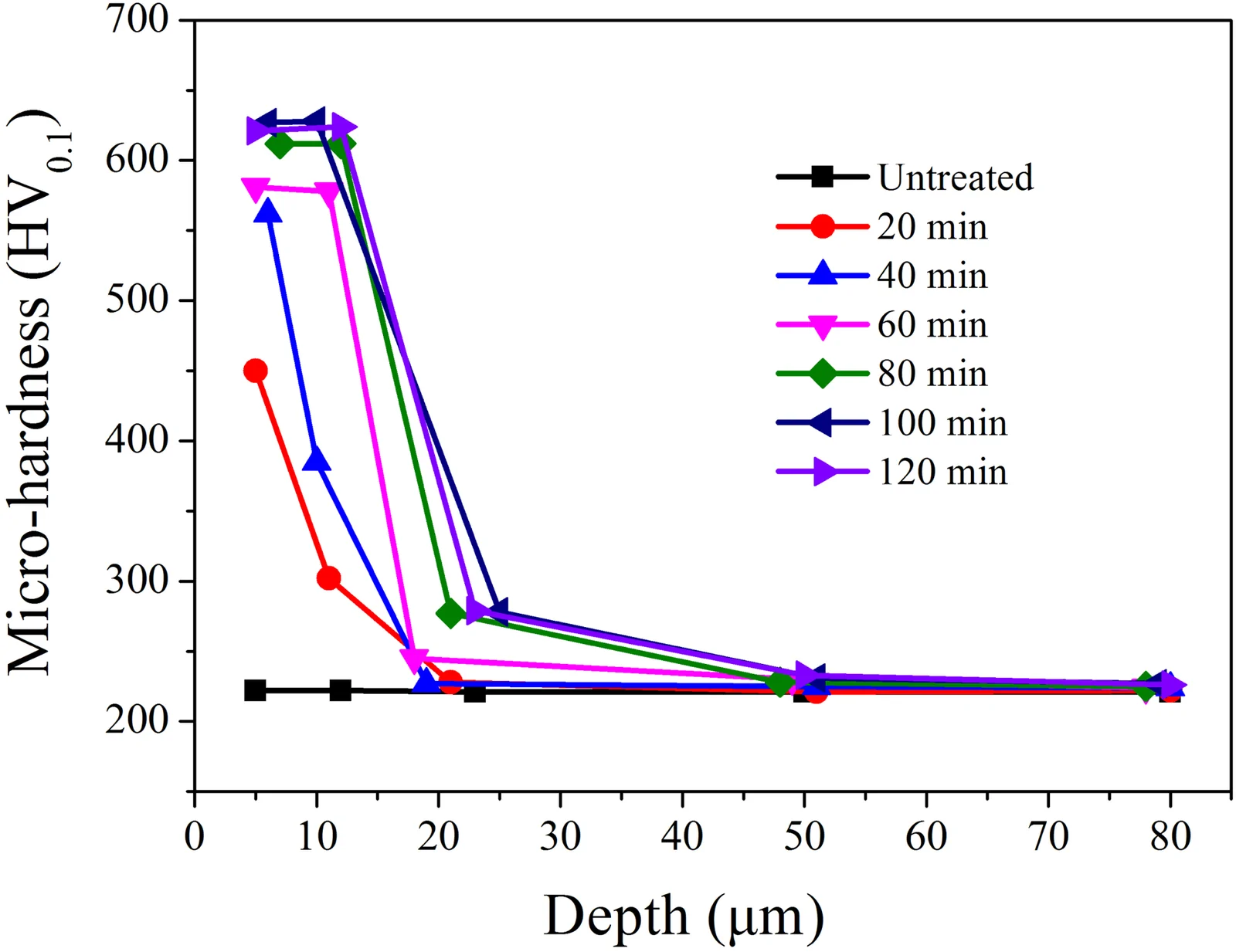
Micro-hardness *vs.* depth plots of the samples nitrided at 500 °C for different times.

As a function of depth, the micro-hardness profiles on cross-sections of the nitrided samples with V_2_O_5_ and untreated sample are shown in Fig. 5. The micro-hardness values of the untreated samples were almost similar in all the measured regions. For the nitride samples with V_2_O_5_, it can be observed that high micro-hardness values are obtained at the nitride layers and then dropped abruptly at the nitride layer/core interface. The depths at which the micro-hardness values are dropped rapidly agree well with the decreasing intervals of nitrogen concentration in the nitrogen concentration profiles (see Fig. 2a), suggesting that the effective thickness of nitride layers can be estimated.

The maximum micro-hardness values on the cross sections of all the samples tend to increase nonlinearly with treatment time. Comparing Fig. 1 and 3 with the maximum micro-hardness values, it can be inferred that the micro-hardness of the nitride layer is more dependent on the composition and crystal structure than the thickness of the nitride layer. About the samples treated for 20 and 40 min, the thickness of the nitride layers is similar, however, there is a significant difference in the maximum micro-hardness values, which can be explained by the different composition and crystal structure of the nitride layers (see Fig. 3). The maximum micro-hardness values for the 40 and 60 min treated samples are not significantly different because the composition and crystalline structure of the nitride layers are almost the same. Only slightly different profiles are obtained due to different thickness of nitride layers. The maximum micro-hardness values of the samples treated for 80, 100 and 120 min are almost similar. However, the maximum micro-hardness value of the sample treated for 120 min were slightly reduced despite the higher nitrogen concentration of the nitride layer and the more advanced structure of the Fe_3_N phase. This can be explained by the surface brittleness caused by the pores and cracks in the nitride layer with increasing nitriding time. Therefore, the reduction of nitriding time by promoting nitriding can be considered as an important factor to ensure the quality of nitride layer.

### 3.4. Wear resistance analysis

**Fig. 6:**
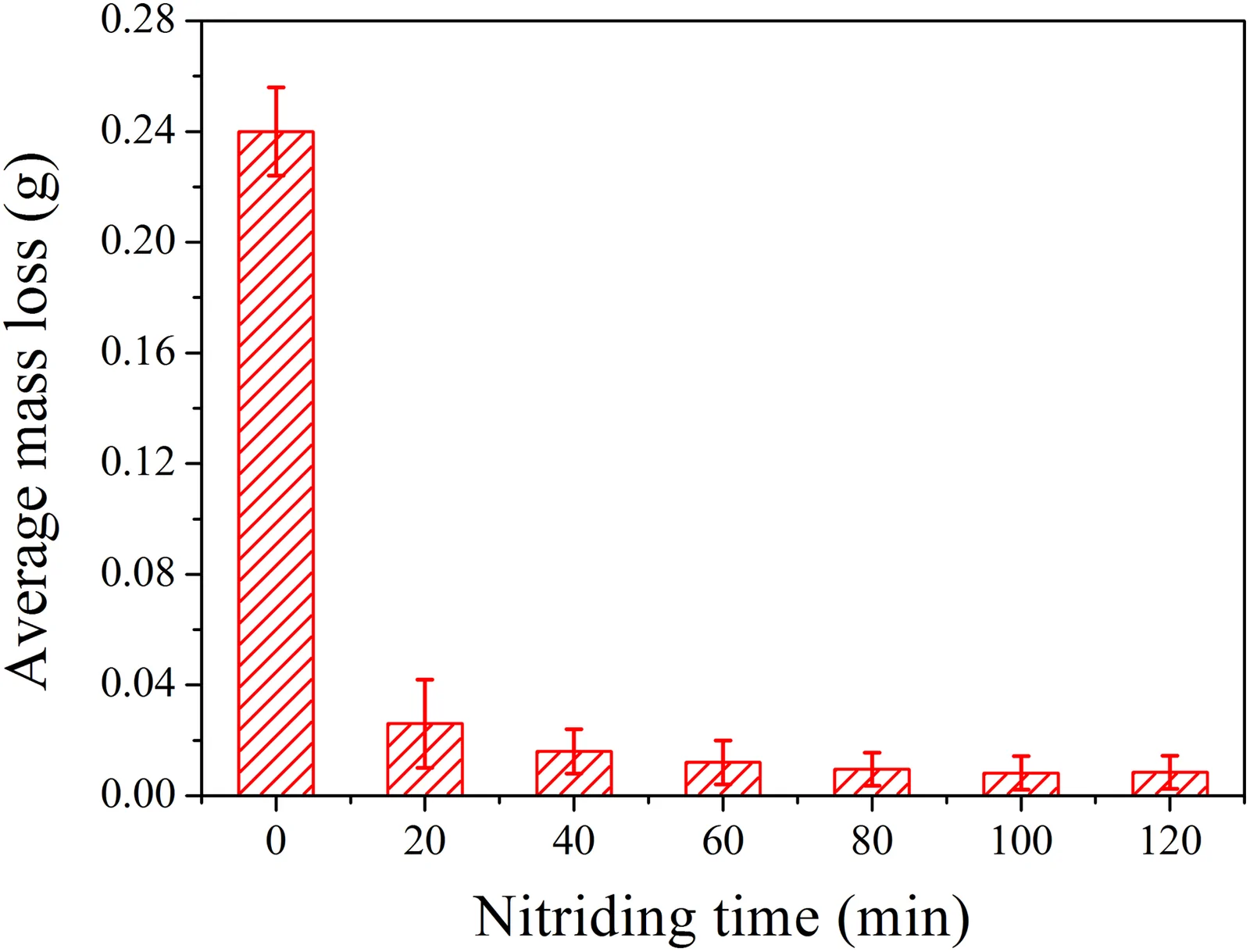
Average mass loss after wear tests for the samples untreated and nitrided for different times.

To investigate the influence of nitriding time on wear resistance of the nitrided samples with V_2_O_5_ and untreated sample, a MMW-1A Wear Test Machine was employed. The average mass loss calculated by three parallel experiments under the same condition is recorded for untreated and treated samples as shown in Fig. 6. It can be seen that the average mass loss in the untreated sample is around 0.24 g.^26^ Through the tests, the average mass loss of the nitrided samples is much lower than that of the untreated sample. With the extension of nitriding time, the average mass loss of the nitrided samples reduces gradually, reaching the minimum of 0.0082 g for the sample nitrided for 100 min. This phenomenon is related to the surface microstructure, composition and thickness of the nitride layers in the samples. As can be seen in Fig. 3, the surface layers of the nitrided samples with V_2_O_5_ are made of Fe_3_N and Fe_4_N with high hardness, which results in excellent wear resistance compared to the untreated sample. The average mass loss of the nitrided samples is in relatively good agreement with the change in the composition of the nitride layer shown in Fig. 3. The average mass loss of the sample nitride for 20 min is enlarged relatively, which could be attributed to the insufficient development of the nitride layer.

### 3.5. Corrosion resistance analysis

#### 3.5.1 Neutral salt spray test

**Fig. 7:**
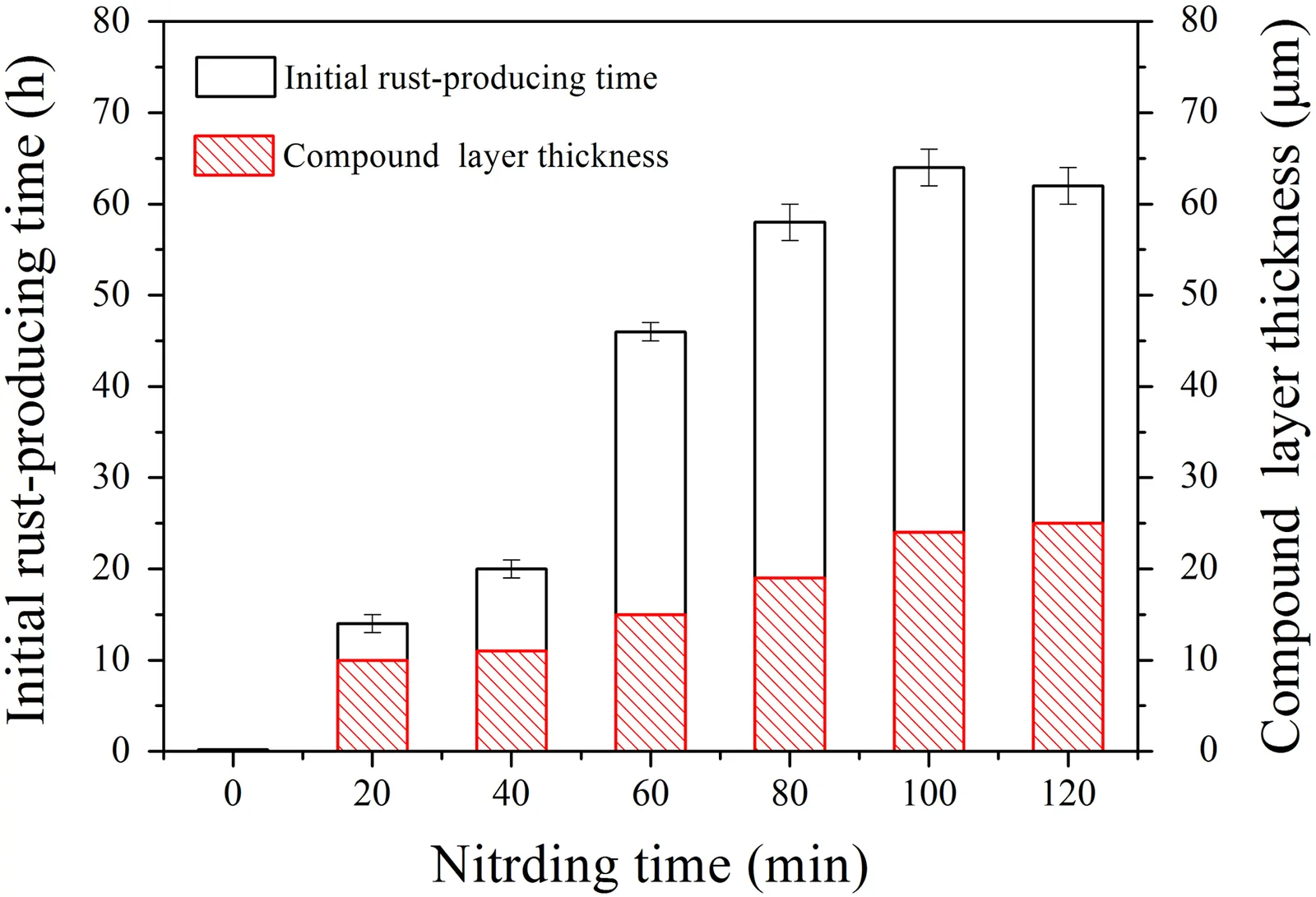
Initial rust-producing times for samples nitrided at 500 °C for different time.

In order to evaluate the corrosion resistance in seawater, a neutral salt spray test was performed for the treated and untreated samples. Fig. 7 shows the initial rust-producing times for the samples treated with different nitriding times. The thickness values of the compound layers obtained from the secondary electron images and microhardness profiles on the cross-sections of the nitrided samples are also shown in Fig. 7. As can be seen in Fig. 7, the initial rust-producing time of the un-nitrided sample is about 12 min, which highlights that carbon steel is not available without suitable surface modification or coating because of its very poor corrosion resistance in seawater. Also, the initial rust-producing times of the nitrided samples are much longer than that of the untreated sample, indicating that the corrosion resistance can be improved by nitriding treatment. This can be attributed to the formation of the nitride layer. With the nitriding time increasing, the initial rust-producing time is increased, reaching the maximum of 64 h when nitrided for 100 min.^44^ It can be inferred that the passage of the corrosive medium through the compound layer of the samples is delayed and the thickness of the compound layer is directly related to the corrosion resistance. However, when nitriding time exceeds 100 min, no significant change in the initial rust-producing time can be observed. It can be seen that the thickness of the compound layers increases with the increasing of nitriding time, but with the development of micro-cracks. Thus, it can be assumed that the corrosion medium is easily accessible to the substrate through these micro-cracks, accelerating the localized corrosion, which resulting in a decrease in overall corrosion resistance. It can be seen that the corrosion resistance of nitrided samples is affected by the thickness and quality of the compound layer. This may be related to the different composition and structure in the nitride layer.

Several mechanisms have been proposed to explain the beneficial role of nitrogen in stainless steel. In those theories, nitrogen stabilizes the film and austenite phase and prevents the attack of Cl^−^ anions. In principle, these theories can also be used to explain the corrosion resistance of nitrided steel. The beneficial role of nitrogen can be attributed to the presence of nitrogen in the active atomic state. This is based on the general knowledge that the beneficial effect of nitrogen can be realized only when nitrogen is in a solid solution. Atomic nitrogen is more active than molecular nitrogen, and therefore can cause various chemical reactions and mitigate corrosion erosion to a certain extent.^23^

#### 3.5.2 Potentiodynamic test

**Fig. 8:**
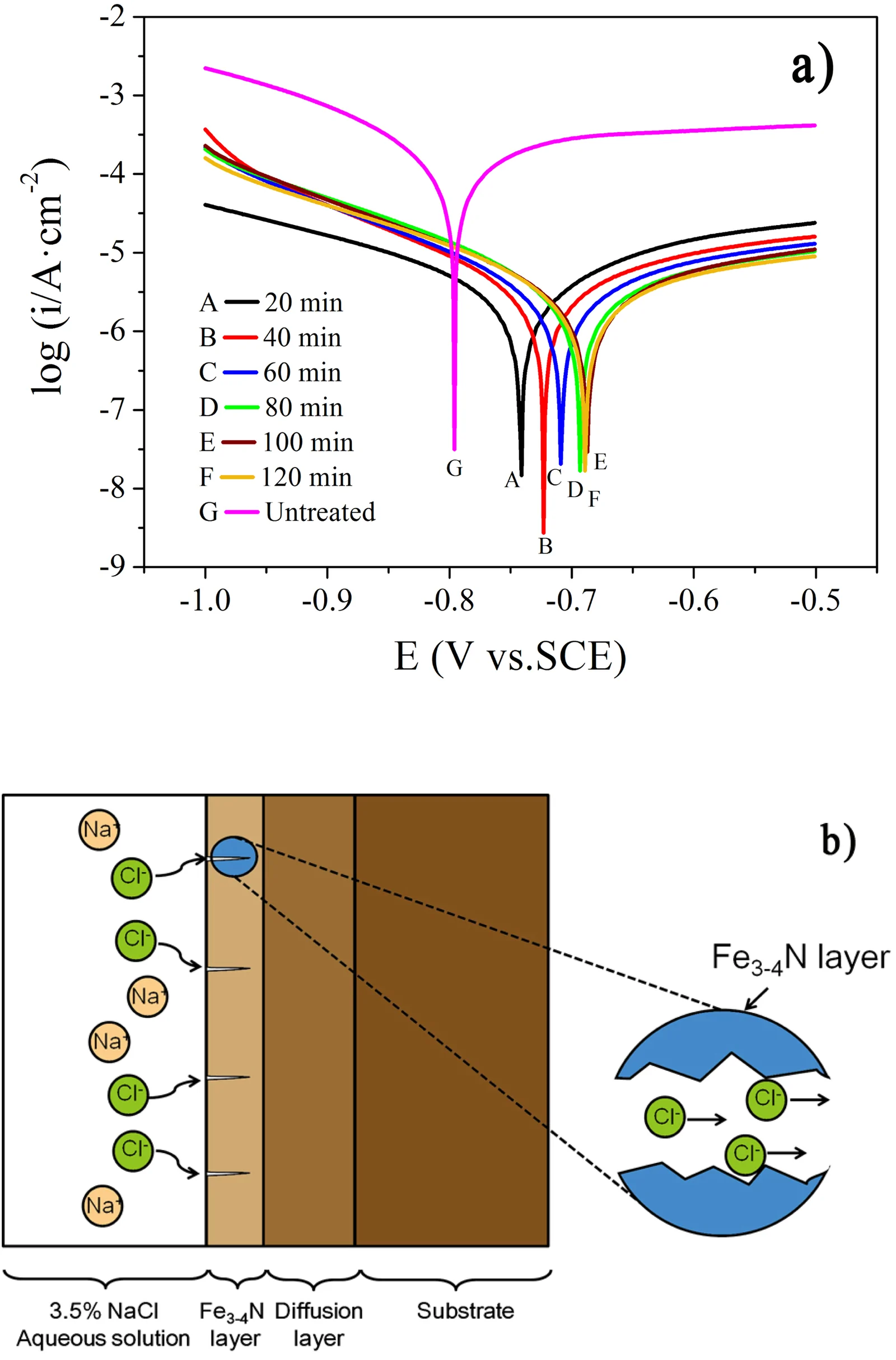
(a) Polarization curves for various specimens at 25 °C in 3.5% NaCl solution (A: 20 min, B: 40 min, C: 60 min, D: 80 min, E: 100 min, F: 120 min, G: untreated) and (b) schematic diagram for the diffusion of corrosion medium.

To investigate the electrochemical behavior of the non-nitrided and nitrided samples for different times, the results of the potentiodynamic test are shown in Fig. 8, where the anodic and cathodic polarization curves are recorded after 30 min immersion in 3.5% NaCl solution at 25 °C. On other hand, the electrochemical parameters calculated from the polarization curves for the untreated and treated samples are listed in Table 1. The polarization curves for the nitrided samples are located on the positive side compared to that for the untreated sample and the corrosion potential is shifted from −0.996 V to −0.687 V, indicating that the anodic reactions are suppressed more than the cathodic ones. Thus, the nitride layers formed during the nitriding process are expected to reduce anodic dissolution and retard hydrogen evolution reactions by protecting the anodes. Also, with increasing nitriding time, the polarization curves of the treated samples tend to shift slightly towards positive, which is in good agreement with the changes in thickness and composition of the nitride layers and the results of neutral salt spray tests. It can be attributed to the well-formed protective barrier by nitride to protect the substrate, as the Fe–N phases are developed and the nitride layers become denser.

**Table 1:** Corrosion parameters calculated from polarization curves of untreated and treated samples

Labels of samples	Nitriding time (min)	Corrosion potential (V)	Corrosion current density (A cm^−2^)	Corrosion rate (g h^−1^)
A	20	−0.741	3.336 × 10^−6^	3.483 × 10^−6^
B	40	−0.723	3.592 × 10^−6^	3.663 × 10^−6^
C	60	−0.709	3.162 × 10^−6^	3.291 × 10^−6^
D	80	−0.693	3.133 × 10^−6^	3.273 × 10^−6^
E	100	−0.687	3.033 × 10^−6^	3.182 × 10^−6^
F	120	−0.689	3.055 × 10^−6^	3.199 × 10^−6^
G	Untreated	−0.996	1.976 × 10^−4^	2.059 × 10^−4^

Compared to the untreated sample, the corrosion current (*I*_corr_) for the nitrided samples is significantly reduced from 1.976 × 10^−4^ A to 3.033 × 10^−6^ A. However, it can be observed that the corrosion current for the nitrided samples decreases slightly with the increase of nitriding time. Similarly, the corrosion rate of the nitrided samples is 3.182 × 10^−6^ g h^−1^, much lower than 2.059 × 10^−4^ g h^−1^ of the untreated sample.^27^ However, it is observed that the corrosion rates of the nitrided samples are gradually decreased with the increase of nitriding time. Considering the results of neutral salt spray tests, the results of corrosion current and corrosion rate are not in good agreement with the changes in the thickness and composition of the nitride layers, unlike the corrosion potential. This suggests that the corrosion current and corrosion rate are also related to the microstructure of the nitride layers. The thickness of the nitride layer can affect the retardation of the diffusion of the corrosive medium, but the effect is reduced when the cracks existing in the nitride layer is widened (Fig. 8b). This could be explained the slight increase in corrosion current and corrosion rate of the sample nitride for 120 min. As a result, the reduction in corrosion current and corrosion rate can be caused by the penetration of nitrogen into the steel surface, indicating that the corrosion performance of the steel substrate can be greatly improved using the nitriding with V_2_O_5_.

#### 3.5.3 EIS analysis

**Fig. 9:**
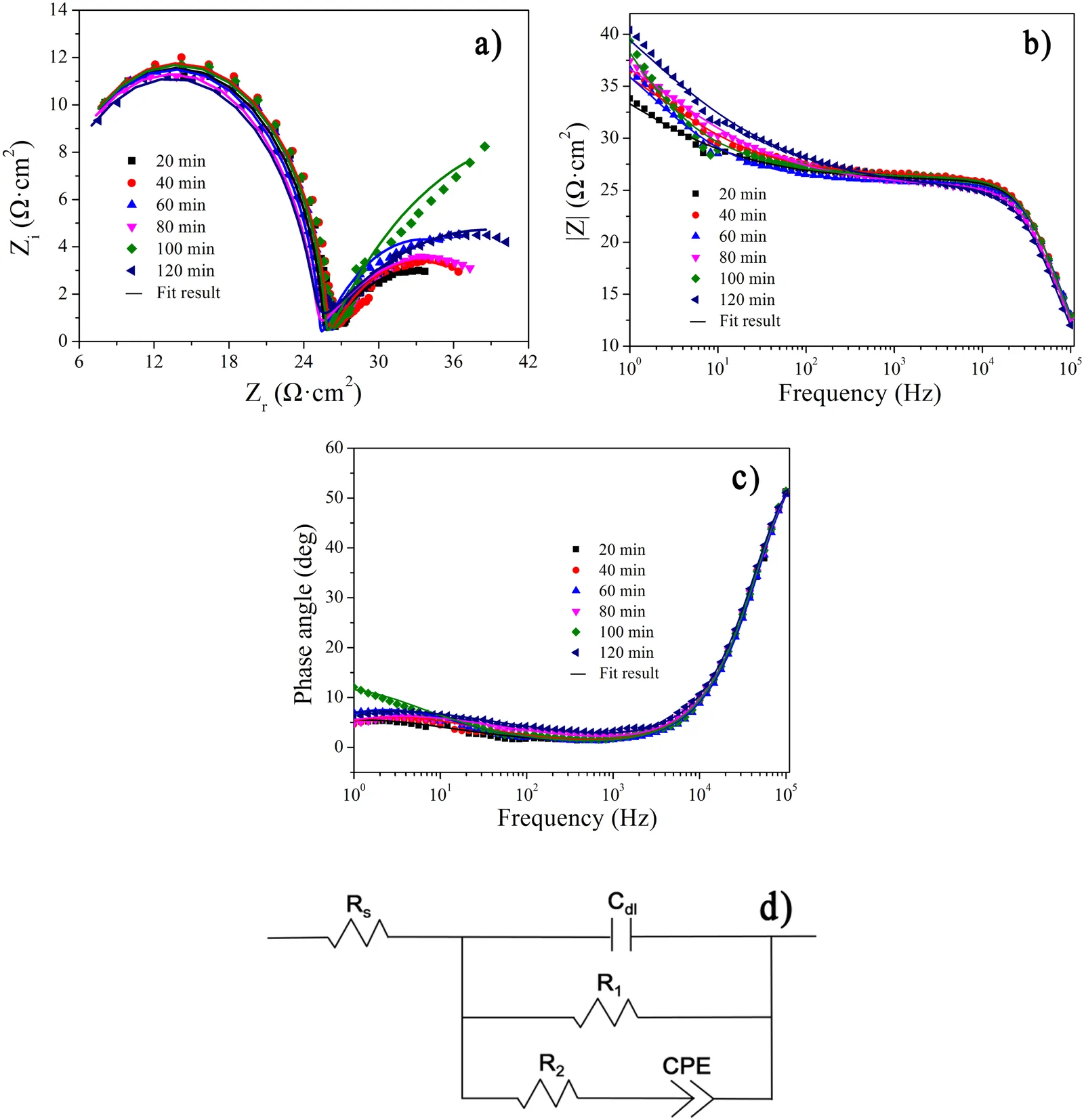
EIS measurement data, fitting curves and equivalent circuit for untreated and treated samples: (a) Nyquist plots, (b) Bode plots of |*Z*| *vs.* frequency, (c) Bode plots of phase angle *vs.* frequency, (d) equivalent circuit.

Electrochemical impedance spectra for the nitrided samples with V_2_O_5_ at its open circuit potential after 2 h of immersion in 3.5% NaCl solution are obtained over the frequency range from 100 kHz to 1 Hz. The shape of Nyquist diagrams for the samples nitrided for different times in 3.5% NaCl solution at 25 °C is similar (Fig. 9a). From these recorded spectra, it can be seen that each impedance diagram consists of two capacitive loops at low frequency and high frequency, which can be attributed to the charge transfer resistance in parallel with the double-layer capacitance. In the studied frequency range, the electrical equivalent circuit employed to analyze the impedance spectra with two capacitive loops is shown in Fig. 9d. In this equivalent circuit, *R*_s_ is the solution resistance, *C*_dl_ is the double-layer capacitance, *R*_1_ is the nitride layer resistance as the resistance of compact inner layer, *R*_2_ is the charge transfer resistance as the resistance of porous outer layer, and CPE represents the capacitive element. In the circuit, the constant phase element, CPE, is introduced to give an accurate fit. The impedance function of a CPE can be expressed as:*Z*_CPE_ = *A*^−1^ (*iω*)^−*n*^where *A* is the CPE constant (in Ω^−1^ s^*n*^ cm^−2^), *ω* is the sine wave modulation angular frequency (in rad s^−1^), *i*^2^ = −1 is the imaginary number, and *n* is an empirical exponent (0 ≦ *n* ≦ 1) which measures the deviation from the ideal capacitive behavior.^24^

**Fig. 10:**
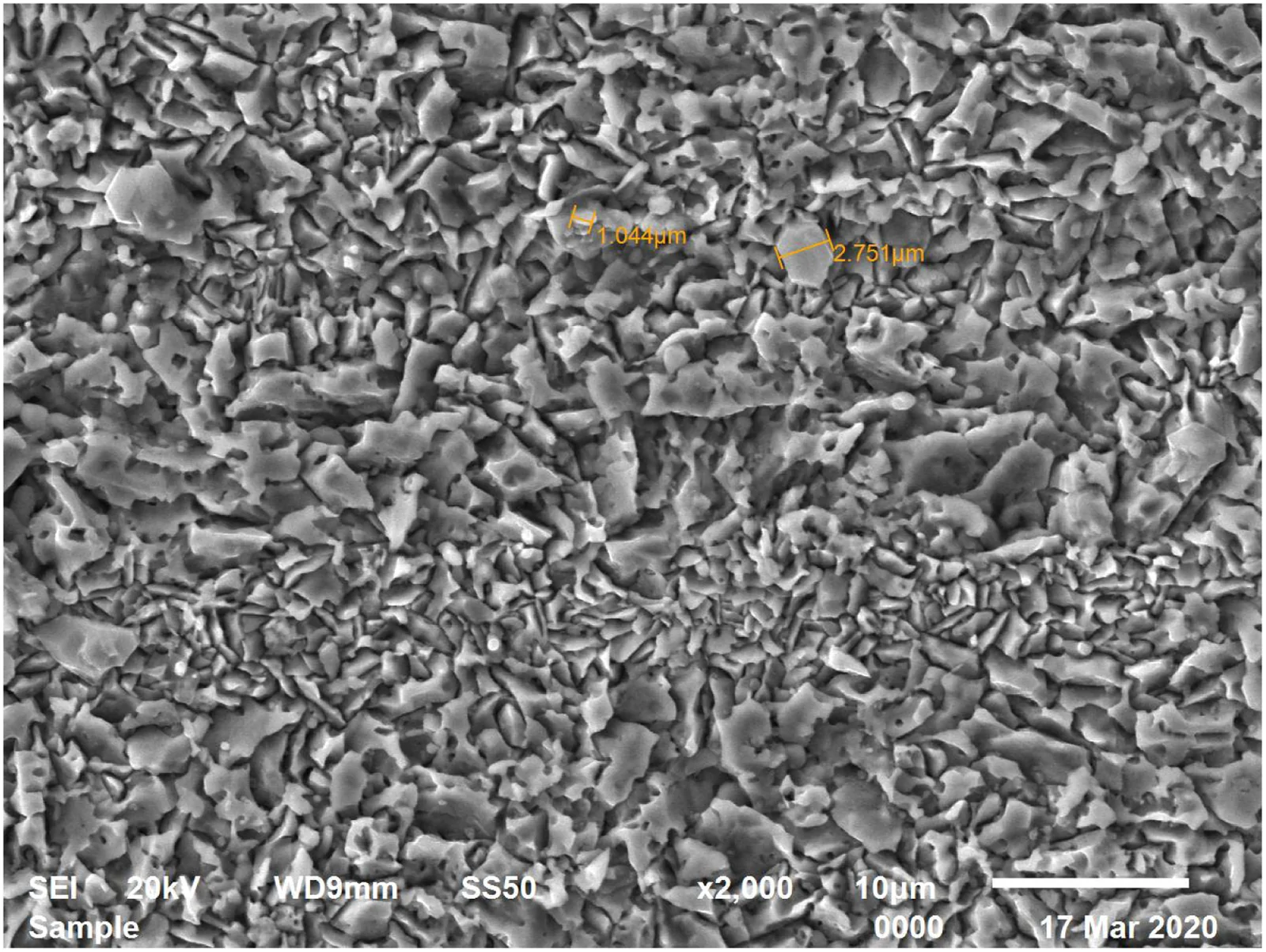
SEM image for the surface of sample nitrided for 120 minutes.

The fitting results obtained by simulating EIS data with the equivalent circuit are listed in Table 2. The data are found to be sufficiently well fitted within the limits of experimental error and reproducibility of data. From the fitting results, the *R*_1_ values obtained for the nitrided samples are gradually increased with the increase of nitriding time, indicating an increase in the resistance of compact inner layers. In contrast, the *R*_2_ values obtained for nitrided samples are gradually decreased, indicating a decrease in the resistance of porous outer layers (Fig. 10). The increase in *R*_1_ value is due to the dense nitride layer with the increase in the thickness of the compound layer on the surface of samples due to the penetration of nitrogen atoms. The decrease in *R*_2_ is related to the occurrence of micro-pores or micro-cracks on the subsurface of the samples due to the generation of nitrogen gas during the subsequent nitriding process. The loose microstructure on the surface of the samples nitrided for 100 and 120 min is considered as the reason for lower *R*_2_ values.^45^

**Table 2:** Fitting results obtained by simulating EIS data with the equivalent circuit

Nitriding time (min)	*R*_s_ (Ω cm^2^)	*C*_dl_ (F cm^−2^)	*R*_1_ (Ω cm^2^)	CPE		*R*_2_ (Ω cm^2^)
				*Y*_0_ (Ω^−1^ s^*n*^ cm^−2^)	*n*	
20	2.591	1.21 × 10^−7^	38.77	0.00458	0.4976	58.7
40	2.554	1.21 × 10^−7^	38.81	0.002371	0.5598	61.3
60	2.489	1.187 × 10^−7^	40.07	0.00254	0.6218	55.38
80	2.487	1.253 × 10^−7^	41.59	0.003223	0.4765	50.31
100	2.581	1.223 × 10^−7^	59.59	0.006709	0.5736	39.13
120	2.384	1.18 × 10^−7^	59.27	0.002987	0.5602	41.36

The values of |*Z*|_*f*→0_ for the nitrided samples are increased gradually with the nitriding time, however, the maximum value of impedances is achieved for the sample nitrided for 120 min (Fig. 9b). It can be seen that the larger the thickness of the nitride layers, the more effectively it limits the penetration of Cl^−^ ions into the substrate when immersed in NaCl solution.

## 4. Conclusions

This study was concerned with the study of surface properties of carbon steel nitrided in a salt bath with V_2_O_5_ and corrosion resistance properties in 3.5% NaCl solution. The surface of the nitrided samples was modified with Fe–N layers composed of Fe_4_N and Fe_3_N, ranging from 10 to 25 µm. Nitriding in V_2_O_5_-added salt bath inhibited the formation of Fe_3_C and promoted the growth of Fe–N phase. The maximum value of micro-hardness in the compound layer was 627 HV_0.1_. The phase composition of the nitride layer had a significant effect on the wear resistance of the nitrided samples. The corrosion resistance of the nitrided samples with V_2_O_5_ was improved significantly compared to the untreated sample. With increase of nitriding time, the corrosion resistances of the nitrided samples were increased non-linearly due to the larger thickness and the looser microstructure of the nitride layer.

## Conflicts of interest

The authors declare that they have no known competing financial interests or personal relationships that could have appeared to influence the work reported in this paper.

## Data Availability

The data that support the findings of this study are available from the corresponding authors upon reasonable request. The results presented in the study have been obtained by using the experiments of electrochemistry.
